# Supplementary material for: Whole and Purified Aqueous Extracts of Nigella sativa L. Seeds Attenuate Apoptosis and the Overproduction of Reactive Oxygen Species Triggered by p53 Over-Expression in the Yeast Saccharomyces cerevisiae
Source: Cells. 2022 Mar 3;11(5):869. doi: 10.3390/cells11050869 (PMC8909299; doi:10.3390/cells11050869)
Supplement: Supplementary file 1 [file cells-11-00869-s001.zip › cells-1563496-supplementary.pdf]

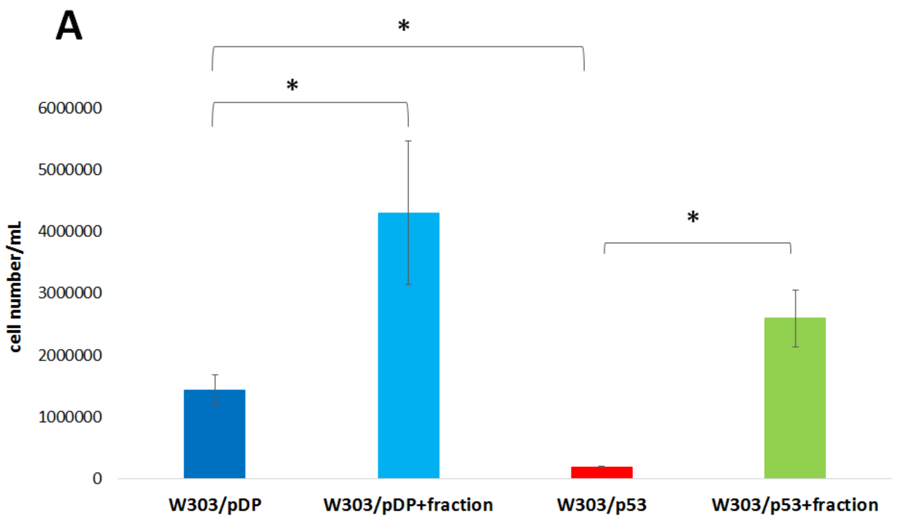

Supplementary Figure S1 : Quantification of the total number of cells by flow cytometry. Yeast cells W303/pDP and W303/p533 cells grown in MMGal with and without C4 fraction were harvested, washed and suspended in PBS. Cell counting was realized by flow cytometry.

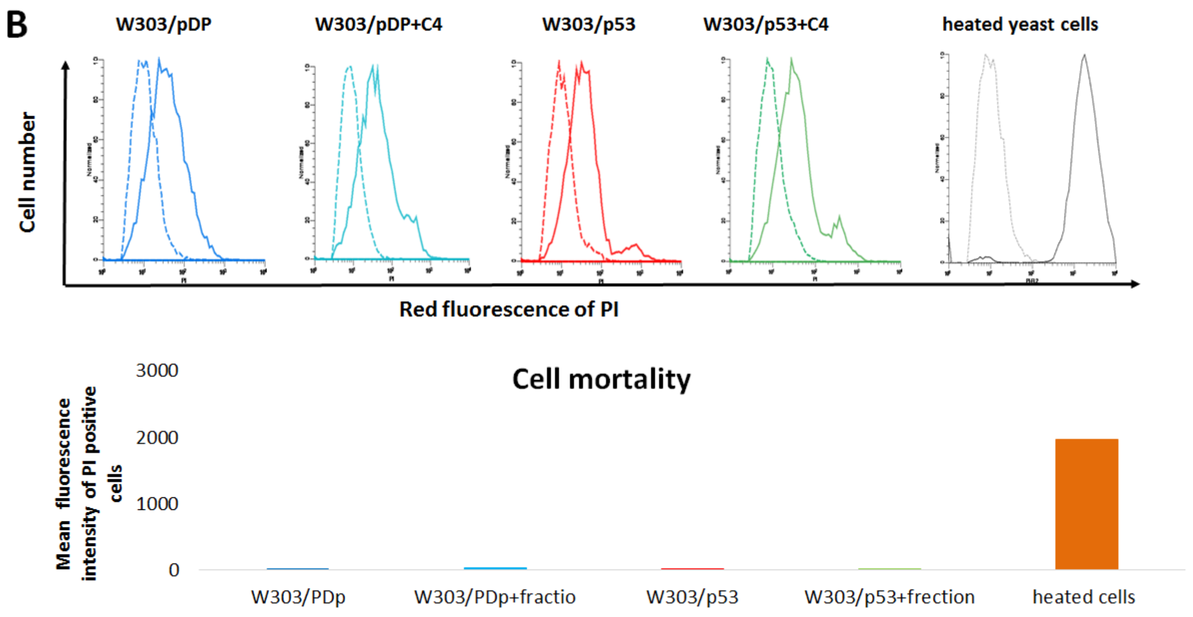

Supplementary Figure S2 : Flow cytometric evaluation of cell death after staining with propidium iodide (PI)**.** Cell death was determined by the mean intensity fluorescence of PI. in both W303/pDP and W303/p533 cells grown in MMGal with and without the C4 fraction, cell death was evaluated by staining with PI. Histograms of stained cells (continuous line) and unstained cells (dotted line). Cell death is estimated by the difference of the mean intensity fluorescence between stained and unstained cells; no difference were observed in the different types of cells with or without C4 fraction. Induction of cell death induced by heating is associated with an important increase of the mean fluorescence of PI positive cells.
